# Medical Institute of Louisville

**Published:** 1844-02

**Authors:** 


### MEDICAL INSTITUTE OF LOUISVILLE.

We expect to be enabled to announce some important changes in the organization of this institution in our March number. Dr. Cooke, the venerable professor of Theory and Practice, having signified to the Board of Managers his determination to retire from the school, at the close of the present session, the Board contemplate reducing the number of chairs to seven. Professor Cooke has been confined to his house in the country, for many weeks, with severe indisposition. He is now, however, we are happy to be able to inform his many friends, convalescent. His complaint was pleurisy. Y.

